# Society Proceedings

**Published:** 1898-10

**Authors:** 


### SOCIETY PROCEEDINGS.

### NEW YORK STATE VETERINARY MEDICAL SOCIETY.

The annual meeting of the society was held Wednesday and Thursday, September 14 and 15, 1898, at the Hotel Metropole, New York City. The invitations extended by the Committee of Arrangements to veterinarians not members of the society brought a great many visitors who were interested in the papers and discussions; in fact, several of them took active part in the debates. The accommodations at the Hotel Metropole were first-class. The meeting-room, well lighted and ventilated, is capable of seating about one hundred and fifty.

The meeting convened at 11 A.M., Dr. Robert W. Ellis, Secretary of the Veterinary Medical Society of New York County, welcomed the members of the State Society in a very neat and appropriate address:

"Although I have not the honor of being a charter member of this Society, it is not because I did not feel as keen an interest in its organization as I have since felt, and now feel, in its successful perpetuation; for, although not able to be present at its organization in Rochester in 1890, or ever able to attend any of its subsequent northern meetings to apply for membership, when the Society met in this city in 1895 I immediately filed my application and became a member, and in 1896 I was pleased to be able to answer the roll-call in Buffalo, and although I could not meet you in Syracuse last year, to-day I have the honor to welcome you to our city in behalf of the Veterinary Medical Association of New York County, and we desire to extend to you, gentlemen, a hearty wholesouled welcome, just such a welcome, in fact, as each one of us in our domestic circles would extend a visiting parent to our homes. For what other, in truth, is our county association than an offspring of the State organization and a grandchild of the National body? For was not the idea of the organization of a county association conceived at a happy moment in the mind of the county secretary for this county of the State Society, who immediately put his idea into execution by calling a meeting of organization, and that meeting of organization was presided over by the President of the United States Veterinary Medical Association at that time, Dr. W. Horace Hoskins, of Philadelphia, Pa.; so you see that establishes our relationship to the National body. Now, while this feeling of relationship to the State Society naturally adds to our inclinations to welcome you to our field of action, it seems to me that it should also act as a stimulus to every member of the Veterinary Medical Association of New York County as well as to every veterinarian of Greater New York, whether a member of the county organization or not, to seek a closer relationship to the State Society by seizing the golden opportunity that this home meeting affords them to identify themselves with this valuable organization. Many members of the County Association are members of the National Association, and by becoming members of the State Society they supply the missing link that binds together the veterinary profession of the continent—County, State, and National. The County Association has indulged in veterinary legislation to some extent, the jury exemption bill having been conceived by the Chairman of the Judiciary Committee, Dr. O'Shea, also an active member of the State Society, and through his untiring efforts it succeeded in becoming a law. It is in veterinary legislation, gentlemen, that a fusion and harmony of the efforts and interests of the State and County organizations can be of incalculable worth to the veterinary profession of the State, and nothing would be more conducive to that condition than an interlacing, as it were, of the State and County organizations through membership in both, and it is therefore that I urge every veterinarian present from Greater New York not already a member of the State Society to take advantage of this opportunity of becoming so; and, perhaps, it would not be out of place if I should take this occasion to add, also, for the benefit of those who have not already availed themselves of the advantage of membership in the County Association, that the monthly meetings at the Academy of Medicine will be resumed on the first Wednesday in October, and owing to the peculiarly favorable situation, surrounded as it is by the several boroughs of Greater New York, there is practically no limit to its possibilities and educational advantages with the increase in membership made possible by such surroundings. In conclusion, I would add that if we can but make you feel the welcome that we would extend to you in the same degree we mean it, and that we realized it during our sojourn among our northern neighbors in Buffalo in 1896 (last year I was out of it), our efforts will not have been in vain. Now, gentlemen, I feel sure we will not have completed the work laid out for to-day before the Committee of Arrangements will be around to advise us that the time for the recreation part of the programme has arrived, unless we cut our addresses short, so I will close my remarks with thanks for your kind indulgence."

On behalf of the members, Dr. James Law responded:

"Dr. Ellis and Gentlemen of the New York County Veterinary Medical Society: It is my pleasing duty to accept, on behalf of the New York State Veterinary Medical Society, the welcome which has been so kindly and generously offered. I assure you, sir, that the pleasure in the reunion is mutual. Your welcome has been most generous. We could expect nothing less. It comes to us from the largest and most important county society in the State, a society which is not simply a county society, as it modestly assumes, but which, as representing Greater New York, may justly claim to be the metropolitan society of the continent. And yet our welcome comes not from strangers, but from friends, not from outside, but largely from within our own ranks, for much of the bone and sinew of our State Society are members of the New York County Society. This common interest and membership is as it should be. The statute which regulates the practice of veterinary medicine in the State tends to unite in one all legally qualified practitioners in the Commonwealth. They may hail from different schools in Europe or America, or Canada, Massachusetts, New York, Pennsylvania, Ohio, or any other State, yet the common sources of their license to practice binds them in one fraternal bond. This bond is their charter of rights, conferring upon them privileges which are not possessed by the graduates of their own respective alma maters who practise outside of the limits of the State. We feel, therefore, that our welcome comes from the ranks of our own State brotherhood, and that it is given and received in the true fraternal spirit. None the less, we take it as a handsome compliment and an earnest of the success of our assembly that the county society as an organization should thus offer us at the outset the hand of its friendship, the indorsement of its countenance, and its valued assistance in our deliberations. To the practitioners of Greater New York who are not members of the county society, but who have honored our meeting by the indorsement of their presence, we would also offer our thanks for their silent welcome, shown in act rather than in word, and we trust that they will carry the same generous spirit further and contribute freely to the success of our deliberations and discussions. The obligations resting on this State society in connection with the administration of the law which regulates the practice of veterinary medicine, and especially as regards the duty of nominating candidates for the position of State veterinary examiners, affect every practitioner in the State, and furnish a strong argument why he should be an active member of the State society. The legal obligation is restricted to the State society, but the moral obligation presses equally upon us all, and just as the resident in a country who profits by the protection of its laws is under a sacred obligation to perform all the duties of the citizen, so the veterinarian practising in this State cannot rid himself of the sacred trust reposed in him under the terms of this State law. If it is the duty of the permanent resident to acquire and wisely exercise the rights of citizenship for the public good, so it is the duty of the veterinary practitioner to qualify himself to exercise his legal rights for the benefit of his profession and of the public at large. It is in view of such rights and duties that in accepting, on behalf of the society, of the very hearty welcome to this metropolitan city I wish to reciprocate by inviting all veterinarians of the State first to help to make the present meeting a success, and, second, to join the State society and help it to wisely perform those duties which are devolved upon it by the statutes."

After the reading of the minutes, President Baker made his annual address. This was followed by the Secretary's report and the reports of all committees. Nomination of candidates to fill the vacancy in the State Board of Veterinary Medical Examiners was then in order, and very promptly Dr. George Berns, of Brooklyn and Dr. W. L. Baker, of Cortland, N. Y., were unanimously elected and recommended to the Board of Regents. The report of the county secretaries followed and brought out some lively discussions, principally on tuberculosis.

On the evening of the 14th, at the invitation of the County Association, the members were given an illuminated trolley ride from the Brooklyn Bridge to Coney Island, where the Bowery was "done" in great shape, the members arriving home sometime about midnight. The next morning they very much enjoyed a drive through Central Park and back by the way of Riverside Drive.

The next in order was the reading of the papers, and with the exception of the one on "The Horse's Mouth," by Dr. F. C. Grenside, were read as per programme. After the President had announced the committees and essayists for the next regular meeting, the selection of the next annual meeting-place terminated in Ithaca, Binghamton, Syracuse, and New York being voted for, and after considerable debate the members were obliged to go to the Secretary's desk and deposit their ballots with the teller, the result being in favor of Ithaca, with the understanding, however, that if the United States Veterinary Medical Association should meet in New York the meeting shall be in conjunction with the same. The Committee on Resolutions then presented several resolutions, including one on the death of our late member, Dr. Thomas Giffen, after which one of the most successful, best arranged and attended meetings adjourned.

*Notes taken at the Annual Meeting of the New York State Veterinary Medical Society.*

THE New York State Veterinary College was well represented. Seven of the eleven papers read were by teachers from that school, and three of the papers were rendered more interesting by the exhibition of charts. Dr. Williams exhibited a very rare and valuable specimen of odontoma.

"The New Treatment for Milk-fever" turned out to be a somewhat imperfect translation from an article in a foreign journal, and a rather defective application of the same to four cows. It struck some that when a title is given as above one would expect something originating with the writer, and not a few were surprised when they found out it was a copy, and one member, Dr. Williams, was obliged to correct some errors, probably due to faulty translation.

Dr. L. McLean, of Brooklyn, entered into nearly all discussions with his usual aggressiveness.

During a discussion on osteoporosis some remarks made by Dr. Williams brought forth a query from Dr. Bell: "Do you (Williams) believe that there is such a disease as rheumatism in horses?" Answer: "All of the cases that I call rheumatism turn out to be something else, and one particular case I diagnosed as rheumatic developed into a very acute case of glanders."

Mrs. Baker accompanied Dr. Baker, of Cortland, and enjoyed a ride through Central Park.

It was gleaned from more than one source, and from those who ought to know, that Cornell contemplates establishing a veterinary department in New York City.

Many were very much surprised when Dr. Baker, President, recommended and distributed during the meeting samples and circulars of an antiseptic preparation called "antiseptin."

### VETERINARY MEDICAL ASSOCIATION OF NEW YORK COUNTY.

THE regular monthly meeting was called to order at 8.45 P.M., Wednesday, October 5th, in Room 37, New York Academy of Medicine, by President Robertson. The roll-call met with few responses, owing to the very uncertain condition of the weather in the evening, and many members having been exposed to the same condition of the elements during the afternoon. The following, however, were present: Drs. Robertson, Gill, Hanson, MacKellar, and Ellis.

The minutes of the June meeting were called for by the President, and were read and approved. There were no committee reports ready, so the President passed to the next order of business, and called for papers. No essayist having been appointed for this meeting, President Robertson took the initiative in "case reports" by describing a very interesting case of "Keraphylocele" with which he had been dealing. A very interesting discussion followed and finally drifted into a general discussion on several topics.

Under the head of new business, the question of the U. S. V. M. A. coming to this city was brought up, but President Robertson thought it better to delay its discussion until a fuller meeting.

At 10 P.M. it was regularly moved and seconded that the meeting adjourn, which was carried by vote.

ROBERT W. ELLIS, D.V.S.,
Secretary.

### NORTH CAROLINA VETERINARY MEDICAL ASSOCIATION.

A MEETING of this Association was held in Wilmington, July 30, 1898. The President, C. R. Ellis, delivered a very interesting address, urging the members to aid in having our bill on proper sanitary regulations passed at our next Legislature. A petition was presented to the Association by the Secretary, with the signatures of all the best M.D.'s in North Carolina, asking our next Legislature to enforce strict inspection of animal foods, and we feel sure of winning with such coöperation.

Drs. W. H. Morris, Elizabeth City; H. G. Lambert, Asheville; and J. M. Peden, Winston, were elected members and certificates presented.

A vote of thanks was given the Hibernian Benevolent Society for their hall. Adjourned to meet in December. Place to be decided by the Secretary.

J. W. Petty, D.V.S.,
Secretary and Treasurer.

### SEMI-ANNUAL MEETING OF THE PENNSYLVANIA STATE VETERINARY MEDICAL ASSOCIATION.

It was a happy choice when, in the closing hours of business on March 9th last, the Association yielded to the earnest invitation of members from the western part of the State and decided to hold its semi-annual session in Pittsburg.

No other city in this Commonwealth excepting, perhaps, the "Quaker City," would be so well calculated to interest and amuse the expectant visitor as Pittsburg. Therefore, when the local committee of arrangements announced that a "trolley-ride" had been planned for the guests there was, in the moment, no thought of the meeting; but with thorough abandonment each member and visitor gave himself to the pleasure of the hour. And what can afford more happiness than the greeting of old professional friends? Another year had rolled around in the life-experience of those who gathered at Franklin. Its pleasant memories were still there; but here was another occasion, and the fraternal spirit was intensified. We cannot forget. Like the trysting times in the life of youth are these meetings with those whose lives have but a single aim and object.

But we must not fail to note the progress of the trolley-car. For miles, on either side of densely-shaded avenues, were elegant residences. Here, within a radius of twenty-five miles, are a million people. While a successful agricultural interest makes a prosperous country, so a manufacturing centre makes a prosperous city, and the smoke of her factory fires that hang in dense clouds everywhere is the secret of her greatness. Here was Schenley Park, containing the splendid library, the munificent gift of Andrew Carnegie. Nothing more worthy can any human being bestow than that which will contribute to the intellectual growth and character of a community. Such a gift gains in the lapse of years, cannot be excelled, and no one can say where its good influence will end. Here flows the deep, serene, majestic Allegheny. On either bank a great city, which on first entrance one might easily mistake for another Gotham.

It was in such a city, with so many magnificent parts, that the semi-annual session, 1898, of the State Veterinary Medical Association was held.

The morning having been exhausted in sightseeing, it was decided to have luncheon before opening the session. This was served in the dining-hall of the building, and was a spread from which if any man retired hungry it was his own fault or his incapacity to eat or enjoy good things. If there was any lack, it was in the guests not being able to demonstrate their ability to even make a perceptible opening in the abundance that had been provided by the local committee to satisfy the inner man. No doubt this committee had themselves travelled and knew the peculiar wants and feelings of an itinerant. To say the least, the luncheon was a grand success.

At exactly 1.15 P.M. the meeting was called to order by the outgoing President, Dr. James B. Rayner. With fitting remarks, Dr. Rayner introduced his successor, Dr. George B. Jobson, of Franklin, Pa., who promptly took the chair and opened the business of the meeting.

Roll-call by the Recording Secretary developed the presence of the following regular members: E. E. Bittles, Henry Bowers, Harry Emery, Jacob Helmer, F. F. Hoffman, W. Horace Hoskins, J. B. Irons, George B. Jobson, J. S. Laycock, George Magee, J. C. McNeil, Otto G. Noack, Leonard Pearson, E. C. Porter, James B. Rayner, N. Rectenwald, W. L. Rhoads, A. F. Schreiber, William G. Shaw, James A. Waugh, A. W. Wier.

Visitors present were: E. S. Bayard, representing the *Stockman and Farmer*, Pittsburg, Pa.; Dr. David Martin, McKeesport, Pa.; Dr. Harry E. Dunn, Pittsburg, Pa.; Dr. P. K. Jones, Pittsburg, Pa.; H. A. Fugenbaum, Pittsburg, Pa.; Dr. H. D. Jackson, Sewickley, Pa.; Dr. G. B. Gilmour, Pittsburg, Pa.; Dr. Spohn, Carnegie, Pa.; Dr. A. W. Hinman, Braddock, Pa.; Mr. George W. Laycock, member of Council for Allegheny, Pa.; Dr. C. W. Boyd, Allegheny, Pa.; H. W. Mayer, Pittsburg, Pa.; Dr. W. Ratcliffe, Waynesburg; Dr. L. A. Reefer, Wheeling, Va; W. Rubermahl, Pittsburg, Pa.; Mr. George L. Speil, Philadelphia, Pa.

The minutes of the previous meeting were ordered adopted as read.

Following the reading and approval of the minutes Dr. Jobson, the President, addressed the house as follows: "Gentlement of the Association: Not having been present at your meeting in Philadelphia when I had the honor of being elected President of the Association, I take this opportunity to thank you for this token of your esteem, and also desire to express my appreciation of my friend Dr. Conard's courtesy in withdrawing his name as a nominee for that office in order to make the election unanimous. Your meeting in Philadelphia, I understand, was a very enjoyable one, as well as highly profitable to those who had the pleasure of being present. But I tell you, gentlemen, when I scan the roll of our members, I do not believe this Association could ever have a meeting which would be otherwise than enjoyable and profitable. This membership-roll also shows me that there are several counties and good-sized towns in this State still unrepresented in this Association, containing, I have no doubt, good material for the increase of our membership. And I would say to those veterinarians who neglect to avail themselves of the opportunity to become affiliated with this organization, that they make a mistake. There are advantages connected with this membership which a new member does not realize. You get acquainted with the best class of your fellow-practitioners, men of recognized ability in the profession; learn new and improved methods of operating and of treating diseases; hear discussed questions of vital interest, bearing on the advancement of the profession. And, let me tell you, whatever puts our profession on a higher plane helps the individual member, and last, but not least, the city member is brought into closer fellowship with his country brother (the men in the field), with mutual benefit to both. What we want is good, solid membership; enthusiastic, united, and willing at all times to work for the good of the profession. This is a progressive age. Is the advance in veterinary science commensurate with the advance in other scientific professions? The introduction of anæsthesia and the use of antiseptics in surgical operations marks a new epoch in human surgery, proving of incalculable value to suffering humanity: the one in rendering the patient unconscious during an operation, the other in reducing the death-rate from septicæmia. Although the majority of our profession fully appreciate the benefit to be derived from antisepsis, probably we do not fully realize the comfort and safety to the patient and operator in using anæsthesia during major operations while the former is secured in recumbent position. The introduction of local anæsthesia has proved a real boon to veterinarians, enabling quite a number of operations to be performed easily and safely which, in former days, it would have been impossible to perform without throwing the animal. Hypodermatic and intravenous injections of the active principles of drugs, in their quickness and efficiency in combating colics and other acute diseases, mark another era in veterinary progress. All these are evidences of progress. But the germ-theory of disease, or the discovery that those epizoötics which spread with lightning-like rapidity over a large extent of territory, as well as other infectious diseases of slower but more insidious growth, are due exclusively to the invasion of the animal economy by specific bacteria in each particular disease, is without doubt the greatest discovery in veterinary science. Following on this discovery came the question of the possibility of transmitting some of these diseases to man through the consumption of the flesh or milk of diseased animals or by accidental inoculation, and the efforts made by the Bureau of Animal Industry and the various livestock sanitary boards to warn the public against these dangers, as well as to control the spread of infectious and contagious diseases among the livestock of their several States. Foremost among these institutions is the good work done by the board of our own State, of which our worthy fellow-member Dr. Pearson is the active member, and whose tact, ability, and scholarly attainments enable him so well to execute the duties of the responsible office of State Veterinarian. Gentlemen, I now close with the earnest wish that our Pittsburg meeting may prove, like its predecessors, thoroughly successful in every respect; successful in increase of membership, successful in new ideas advanced for the good of the profession, and a success in the good-fellowship engendered among those present as well as the pleasure we derive from this visit to our brother veterinarians of the Smoky City."

At the conclusion of the President's address, the Corresponding Secretary presented a list of the names of those who desired to enter the Association. This list was placed in the hands of the Board of Censors, Drs. Hoskins, McNeil, and James B. Rayner constituting said board. A brief recess was ordered to afford opportunity for the trustees to transact business; also that the Corresponding Secretary might collect the dues.

Dr. Rhoads read a letter from Dr. E. Sturge, of Scranton, in which the latter presented his resignation, having entered upon the practice of human medicine.

Closing its session, the chairman of the Board of Trustees, Dr. James B. Rayner, announced their approval of the following applicants for membership: Dr. Charles Boyd, Allegheny, Pa.; Dr. J. Buckham, Beaver Falls, Pa.; Dr. George A. Dunn, Pittsburg, Pa.; Dr. Philip K. Jones, Pittsburg,

Pa.; Dr. H. S. Jackson, Sewickley, Pa.; Dr. David Martin, McKeesport, Pa.; Dr. W. A. Meredith, Corry, Pa.; Dr. B. C. McClintock, Titusville, Pa.; Dr. C. W. Ratcliffe, Waynesburg, Pa.; Dr. John J. Repp, Philadelphia, Pa. A motion to suspend the rules governing the election of new members was made by Dr. Hoskins, and the Secretary was instructed to cast a ballot in favor of the same. The application for membership of Dr. Laberge was laid over until the spring session. The resignation of Dr. E. Sturge was accepted, he being found in good standing and his dues paid. According to usual custom, the newly-elected members present were intoduced to the Association.

The Corresponding Secretary rendered a brief verbal report, stating that the financial condition of the Association was excellent; that its membership was growing steadily, the last three meetings alone having added thirty new active members. Referring to the educational power and the social advantage of such a growing organization, he urged those who were not already members to add their names to the list.

County Secretaries' Reports: Dr. Harry Emery, of Wilkinsburg, Pa., in his capacity as County Secretary, had communicated with the profession in his county, and while no promises of papers were given, an interest was manifested such as bode good for the reputation of the county at the meeting in Pittsburg. Dr. M. J. Collins, of Myerstown, Pa., reported for Lebanon County that there was a great deal of tuberculosis in that section of the State; also a disease exists in the northern part of the county among poultry, characterized by protrusion of the eyeball, swelling of the head, and a discharge from the mouth. Some of the eyes rupture; others, that otherwise recover, are left blind. Azoturia is reported as prevalent.

The President stated that letters of regret had been received from Drs. C. C. McLean, of Meadville, and R. A. Dunn, of Titusville.

Reports of Committees: At this time Dr. Leonard Pearson gave his hearers a pleasant and interesting verbal sketch of his recent visit to Europe,[1] attending the fourth session of the Congress for the Study of Human and Animal Tuberculosis, held in Paris from July 27th to August 2d.

At the conclusion of Dr. Pearson's talk, there being no new business or any unfinished business, the essays and papers were called.

Dr. F. F. Hoffman read a short but practical and valuable paper on "Cæsarean Section in the Cow." Dr. E. C. Porter was listened to with interest on "Parturient Apoplexy." Dr. Helmer read Dr. J. C. Michener's essay on "Prognostication," also Dr. Phillips' report. Dr. James A. Waugh presented "Dislocation of Metatarsal and Cuneiform Joints." Dr. E. E. Bittles, "Eserine." Dr. S. J. J. Harger not being present, his paper, entitled "Auto-intoxication in Carnivora," was read by Dr. Hoskins. The valuable point in Dr. Hoffman's paper was where and how to make the incision on entering the uterus. The essayist showed that it must never be made across the general direction of the bloodvessels, which would result in profuse hemorrhage and also laceration on removal of the fœtus. If the incision is made parallel with the vessels the bleeding is slight and laceration is largely averted. Dr. Hoffman's paper was discussed by Dr. James B. Rayner. Dr. Porter's by Dr. Irons, who related some novel ways in which cows had been cured of parturient apoplexy by

[1] The paper was published in full, p. 592 September JOURNAL.

methods that to-day are not considered scientific. This created considerable amusement among the members.

Dr. Pearson took advantage of the occasion to speak of the value of auto-intoxication theories to solve the problem of the cause of parturient apoplexy. The elaborate and ingenious theory of Frank, explaining the causes as mechanical, was popular for many years, until the Schmidt-Mulheim theory of auto-intoxication came upon the field. According to this view, toxic ptomaïns, absorbed from the uterus about the time of birth and subsequently, accumulate in the blood in sufficient quantity to produce irritation of the great nerve-centres, thus giving rise to the variety of symptoms and phases observed in parturient apoplexy. This has been a popular theory and has constantly won admirers, although the results of combating the malady by the treatment suggested was not as satisfactory as was hoped. But recently a Danish investigator by the name of Schmidt advanced the same principle, but differing in that the source of the absorbed poison is in the udder instead of the uterus. Old residue and new colostrum undergoing fermentation produce the toxic elements. Schmidt treats by washing out the udder with a solution of two drachms of iodide of potassium in a quart of sterilized lukewarm water. The apparatus necessary consists of a small glass funnel to which is attached a delicate rubber tube, on the extreme end of which is a silver-plated tube. The tube is inserted into one of the teats and one-half pint of the fluid is thus passed into the quarter, and is, by a gentle massage, diffused as far into the udder as possible. The other quarters are treated in a similar manner, and the operation is repeated in ten hours if necessary. It is advised to use adjunct treatment also, according to the symptoms met with in each case. The method is largely used and is popular in Denmark. In this country some favorable reports have been received, although its use here has been of short duration.

Dr. Rectenwald asked questions as to the application of this treatment, and cited two cases of apparent instances of the disease that developed before parturition. Dr. Rayner holds that of all diseases this one must be treated according to the rules of common sense and the requirements of the case; no procedure or remedy can always be depended upon.

Dr. Pearson called for Dr. Jobson to report his experience with a case treated successfully by the new method. Dr. Jobson said the case had been down ten hours when he arrived. It had grown gradually worse. He carefully applied the Schmidt treatment himself after having the udder stripped of milk. But, in addition, he had used a 1 per cent. solution of creolin as an injection into the uterus, since there was a large amount of mucus issuing from that organ. Internally he administered two pounds of Glauber-salts and an injection of one-half pint of crude oil in a gallon of warm water. The animal made a prompt recovery.

Dr. Bittles discussed the subject. Dr. Rhoads called upon Dr. Schrieber to give his experience with the new mode of treatment. Dr. Schrieber did not consider his a representive case and not a good one upon which to test the value of the new method. Dr. Waugh's paper was discussed by Dr. Rayner, Dr. Bittle's paper by Drs. Porter, Noack, Bowers, Rectenwald, and Irons.

Here Dr. Hoffman introduced the subject of anthrax. He employed inoculation successfully, and had seen two cases of carbuncular anthrax in men. Dr. Jobson used vaccine, and considers it a preventive.

Dr. Hoskins returned to the report of the Committee on Legislation. Under this heading, he said the Board of Examiners had investigated some fifty cases in six months. These were settled by the removal of the offenders in some instances and abandonment of the work in others. Moral suasion had been highly successful in these. We have now on hand four or five hard cases which will test the validity of the present law. Three were those of false registration since the law of 1895. In two of these the Prothonotary struck out the registration. One will be contested. In this case the cause was the neglect of the offender to register in time. We are laboring under the disadvantage of a decision by Judge Schuyler to the effect that no law can establish a limit of time to those in legal practice in our own State.

Dr. Bowers: "I should like to ask whether a practitioner must receive compensation in order to be an offender."

Dr. Hoskins: "We have no legal decision on that point."

Dr. Bowers cited a case of a blacksmith treating a horse.

Dr. Hoskins: "I think it hard to convict blacksmiths who treat hoof troubles, especially if they use their own tools, confine themselves to the hoof, and when they do not charge more than for ordinary work. A mistake was made in the old law. The work of registration should have been done by a board appointed for that purpose, instead of by the prothonotaries. This has caused many illegal registrations. In the case of a board there would be no incentive except to register names legally. In one county men were advertised for who would register, and in sixty-eight cases only twelve were legal. In this case the fee had a glaring influence."

It was moved by Dr. Rayner, seconded by Dr. Waugh, that the discussion of Dr. Harger's paper be laid over until the annual session in Philadelphia; carried.

With appropriate remarks, Dr. Hoskins considered the work that had been so well and thoroughly done by the local committees for the care and comfort of the members, and moved that a vote of thanks be tendered to it by the Association for its hospitality; seconded by Dr. Rayner. This motion was unanimously carried.

On motion the meeting adjourned to meet in Philadelphia, March 7 and 8, 1899.

Thus ended the work of a pleasant and profitable session of the Association.

JACOB HELMER,
Recording Secretary.

GEORGE B. JOBSON,
President.

---

Hamburg, Germany, renders through its Board of Commerce an emphatic protest against the exclusion of American meat-products. Freedom from impurities, healthfulness, and cheaper price are reasons strongly urged why agrarian movements against these products should not prevail.

